# Chronic Obstructive Pulmonary Disease and the Cardiovascular System: Vascular Repair and Regeneration as a Therapeutic Target

**DOI:** 10.3389/fcvm.2021.649512

**Published:** 2021-04-12

**Authors:** Srikanth Karnati, Michael Seimetz, Florian Kleefeldt, Avinash Sonawane, Thati Madhusudhan, Akash Bachhuka, Djuro Kosanovic, Norbert Weissmann, Karsten Krüger, Süleyman Ergün

**Affiliations:** ^1^Institute of Anatomy and Cell Biology, Julius-Maximilians-University Würzburg, Würzburg, Germany; ^2^Excellence Cluster Cardio-Pulmonary System (ECCPS), Universities of Giessen and Marburg Lung Center (UGMLC), Member of the German Center for Lung Research (DZL), Giessen, Germany; ^3^Department of Biosciences and Biomedical Engineering, Indian Institute of Technology Indore, Indore, India; ^4^Center for Thrombosis and Hemostasis, University Medical Center Mainz, Mainz, Germany; ^5^UniSA Science, Technology, Engineering and Mathematics, University of South Australia, Mawson Lakes Campus, Adelaide, SA, Australia; ^6^Sechenov First Moscow State Medical University (Sechenov University), Moscow, Russia; ^7^Department of Exercise Physiology and Sports Therapy, University of Giessen, Giessen, Germany

**Keywords:** COPD, emphysema, pulmonary hypertension, hypoxia, oxidative stress

## Abstract

Chronic obstructive pulmonary disease (COPD) is a major cause of morbidity and mortality worldwide and encompasses chronic bronchitis and emphysema. It has been shown that vascular wall remodeling and pulmonary hypertension (PH) can occur not only in patients with COPD but also in smokers with normal lung function, suggesting a causal role for vascular alterations in the development of emphysema. Mechanistically, abnormalities in the vasculature, such as inflammation, endothelial dysfunction, imbalances in cellular apoptosis/proliferation, and increased oxidative/nitrosative stress promote development of PH, cor pulmonale, and most probably pulmonary emphysema. Hypoxemia in the pulmonary chamber modulates the activation of key transcription factors and signaling cascades, which propagates inflammation and infiltration of neutrophils, resulting in vascular remodeling. Endothelial progenitor cells have angiogenesis capabilities, resulting in transdifferentiation of the smooth muscle cells *via* aberrant activation of several cytokines, growth factors, and chemokines. The vascular endothelium influences the balance between vaso-constriction and -dilation in the heart. Targeting key players affecting the vasculature might help in the development of new treatment strategies for both PH and COPD. The present review aims to summarize current knowledge about vascular alterations and production of reactive oxygen species in COPD. The present review emphasizes on the importance of the vasculature for the usually parenchyma-focused view of the pathobiology of COPD.

## Chronic Obstructive Pulmonary Disease (COPD)

Respiratory diseases are a major cause of morbidity and mortality worldwide. COPD is caused by a persistent obstruction of the airflow in the lungs, which has profound effects on cardiac function and gas exchange, with systemic consequences. The condition arises either because of emphysema, where the pulmonary air sacs are damaged, or because of chronic bronchitis, which is characterized by continuous airway inflammation (1). According to the 2017 World Health Organization (WHO) Global Burden of Disease Study, 3.17 million people died because of COPD in the year 2015, and 251 million individuals were reported to have COPD in 2016. Alarmingly, COPD will be the third-leading cause of mortality worldwide by 2030 (2). COPD is most prevalent in low- and middle-income countries. In developing countries, exposure to biomass smoke, especially during cooking, exposure to harmful smokes during work and underlying diseased conditions (such as tuberculosis) acts as trigger during COPD infections (3). Individuals who have high levels of exposure to tobacco, dust, harmful chemicals, and fumes from burning fuel, as well as individuals with alpha-1-antitrypsin deficiency, are more prone to developing COPD (4). In Western countries, long-term tobacco smoking is the main reason for the development of COPD.

The systemic consequences of COPD can initiate various comorbid diseases, such as ischemic heart disease, heart failure, osteoporosis, normocytic anemia, lung cancer, depression, and diabetes (3). On a cellular and molecular level, these changes are initiated by important upstream events, encompassing the influx of leukocytes, an imbalance of proteases/antiproteases, and increased production of reactive oxygen species (ROS) (4–6). COPD is a multifactorial disease; however, the most studied fundamental mediators are oxidative stress, inflammation, and a lack of physical activity. An important COPD-associated pathophysiology is the spillover of pulmonary inflammation into the systemic circulation (7, 8). Inflammation gives rise to neutrophil extravasation (markers include elastase and calprotectin) and production of inflammatory cytokines, including tumor necrosis factor-α (TNF-α), interleukin 1β (IL-1β), interferon α/γ (IFN-α/γ), interleukin 6 (IL-6), interleukin 8 (IL-8), reactive proteins, and leukotrienes. The constant recruitment of inflammatory immune cells encourages neutrophil infiltration into the lungs, thereby activating the release of proteases and free radicals and resulting in decreased lung elasticity (9). Thus, COPD in the lungs is accompanied by destruction of the elastic architecture of the lung parenchyma, leading to the enlargement of distal airspaces (10). In addition to being an airway and systemic inflammatory disease, COPD also appears to be a vascular disease. It is assumed that cigarette smoke (CS) is vasoactive and directly affects the pulmonary vasculature. Consequently, dysfunction of the blood vessels promotes vascular remodeling, pulmonary hypertension (PH), and finally cor pulmonale (11–13).

In patients with COPD who smoke, oxidative stress is elevated due to chronic exposure to CS and other toxic air pollutants (9). Epidemiological studies that have explored tobacco smoke exposure in patients with PH showed that ~49% of these patients were smokers, of whom 71% were male. In females, PH was often caused by second-hand (passive) exposure to tobacco smoke (14). Lungs are a common site for oxidative stress due to their oxygen-rich microenvironment and frequent exposure to environmental toxins and pathogens. Chronic cigarette smoking results in the progression of COPD due to excessive endogenous ROS production, both from dysfunction of mitochondrial complexes I and III and persistent activation of inflammatory cytokines. In addition, ROS-generating enzymes, such as NADPH oxidases, xanthine oxidases, and heme peroxidases, promote the infiltration of inflammatory cells inside the airways (15). Another major pathophysiological characteristic of COPD is an imbalance in protease/antiprotease levels. There are three classes of proteases linked with COPD pathology: serine proteases, matrix-metalloproteinases (MMPs), and cysteine proteases. Serine proteases are mucus stimulators, which exacerbate airflow obstruction. MMPs degrade protein components of the extracellular matrix (ECM), leading to tissue damage and increased macrophage infiltration, while cysteine proteases, which include caspases, stimulate apoptosis in alveolar epithelial cells (16). Thus, the imbalance is due to excessive neutrophil accumulation, which triggers pulmonary dysfunction.

The pathophysiological interlink between vascular disease and COPD embraces conditions such as PH, hypoxia, systemic inflammation, and oxidative stress (17). The severity of disease in PH-associated COPD significantly increases in individuals who also have pulmonary fibrosis or emphysema, with the survival rate decreasing by up to 50% (18, 19). PH in COPD is characterized by a mean pulmonary artery pressure (mPAP) between 21 and 24 mmHg in the presence of pulmonary vascular resistance (PVR≥3 Wood Units) or an elevation of mPAP 25–34 mmHg, with nearly normal cardiac output (20, 21). An increase in mPAP≥ 35 mmHg or a mPAP≥ 25 mmHg with a low cardiac index (<2.0 L·min^−1^·m^−2^) is considered severe PH in COPD. An mPAP of more than 40 mmHg is frequently observed in patients with severe COPD. Since the presence of PH clearly increases mortality, the occurrence of PH in patients with COPD is of important prognostic relevance (18, 19, 22). The exact prevalence of PH in patients with mild or moderate COPD has not been accurately determined. However, the mortality rate is ~30% in cases of heart disease-related COPD (23). Some other published reports have suggested that the occurrence of PH with mild, moderate, and severe cases of COPD is 16–44, 43–56, and 59–84%, respectively (24–26). The incidence of severe PH in patients with Global Initiative for Chronic Obstructive Lung Disease (GOLD) stage IV was reported to be 3–5% (mPAP>35 to 40 mmHg) (21).

PH is suggested to be the result of hypoxia associated with COPD (27). It has been shown repeatedly that vascular alterations often appear before alveolar destruction is detectable (28–31). A decrease in alveolar oxygen tension in COPD results in constriction of the pulmonary arteries, leading to hypoxia in the body. Elevated alveolar hypoxia is not restricted to smokers with COPD, however, there have been few reports demonstrated that smokers who had not diagnosed COPD also exhibited conditions of cor pulmonale. A study performed using the C57BL/6 mouse model showed that tobacco smoke induced emphysema, promoted remodeling of pulmonary vasculature, increased airspaces (which included changes in parameters such as the surface area and volume of the alveolar walls/septa), and decreased the number of alveoli; this was accompanied by alterations in lung compliance, tidal volume, and airway resistance (29). Drugs such as tadalafil (a phosphodiesterase type 5 inhibitor) and piclamilast (a phosphodiesterase type 4 inhibitor) have been reported to prevent CS-induced emphysema in a mouse model by improving pulmonary performance, lung tidal volume, pulmonary vascular remodeling, systolic pressure, and hypertrophy of the right ventricle (27). The present review summarizes the state of current knowledge about vascular alterations that occur in COPD.

## Causal Role of PH For Right Ventricular Failure in Patients With COPD

PH in COPD is slowly progressive, and mPAP can often remain stable over a period of 3–12 years (32–34). It has been shown that the average change in mPAP can be just+0.5 mmHg/year, independent of the presence of initial PH (defined by mPAP>20 mmHg) (34). Another study that investigated the pathobiology of PH in COPD over time (initial mPAP <20 mmHg) demonstrated that only 33/121 patients developed PH after 6.8 ± 2.9 years (35). However, ~30% of patients with severe COPD exhibited a remarkable worsening of mPAP during follow-up. According to WHO, COPD patients with PH are categorized as Group 3 PH. Group 3 PH patients have a significantly lower survival rate in comparison with PH patients without COPD (Group 1), followed by Group 4 (patients with chronic thromboembolic PH) and Group 5 (patients with hematologic disorders, systemic disorders, and metabolic disorders) (36). A population-based study reported that a higher proportion of older males (aged more than 70 years) are prone to PH with COPD. This population also suffered from co-morbidities such as diabetes, hypertension, coronary artery disease, and atrial fibrillation. In addition, patients with Group 3 PH showed increased left ventricular mass and end-diastolic diameter. The prevalence of PH in patients with COPD depends on the definition of PH, the severity of COPD, and the mPAP, which ranges from 20 to 91% (37). Most patients (90%) with PH have mPAP>20 mmHg, with the majority varying between 20 and 35 mmHg. These patients were characterized by a progressive worsening of partial oxygen/carbon dioxide pressure (PaO_2_/PaCO_2_) over time. In addition, there was an association between alterations in PaO_2_ and mPAP (33, 34). Pulmonary anatomic changes can result in respiratory failure, both type I, where PaCO_2_ <45 mmHg (6 kPa) i.e., normal or low and the partial pressure of oxygen, PaO_2_, is low (hypoxemia); and type II, where PaCO_2_>45 mmHg (6kPa) and PaO_2_ <60 mmHg (8kPa) (38, 39). Within 5 years of diagnosis, 7% of patients with COPD will experience hypoxemia. The pathology of inpatients with right heart failure (RHF) is commonly preceded by PH. The severity of PH and the development of RHF are closely associated. PH increases the workload of the right ventricle, leading to hypertrophy, dilatation, and ventricular dysfunction. RHF is frequently accompanied by peripheral edema and can be observed in patients with advanced COPD (40, 41). Peripheral edema is considered to reflect RHF, but the possible occurrence of RHF is sometimes assumed to simply indicate the presence of secondary hyperaldosteronism induced by functional renal insufficiency (42).

The effect of pressure overload in the development of RHF has been intensively discussed, probably due to additional causes independent of PH. In patients with stable COPD, right ventricular contractility, measured by the end-systolic pressure–volume relationship, is not abnormal in COPD patients suffering from PH. Notably, many patients with advanced COPD never develop RHF. The level of mPAP is suggested to be a valuable prognostic indicator for patients with COPD (39, 43). Accordingly, life expectancy is less in patients suffering with PH compared with patients that do not have PH (18, 44, 45). The 5-year survival rate of COPD patients with PH (mPAP>20 mmHg) is about 50%. In PH, there is an increased mean pulmonary arterial blood pressure (46) that causes an increased afterload for the right ventricle (RV) of the heart leading to right heart hypertrophy. This adaptive hypertrophy helps the heart to deal with the high pulmonary vascular resistance (PVR). However, this beneficial adaptive hypertrophy can result in maladaptation, RV dilatation and finally failure. The term “cor pulmonale” was used to define a right ventricular dilation due to COPD. However, present studies describing patients with mild-to-moderate COPD demonstrated reduced RV volumes compared with healthy controls (47). This discrepancy can be explained by the fact that majority of the patients with severe COPD primarily suffer from increased intrathoracic pressures due to hyperinflation and airway obstruction, but not from right heart failure. Indeed, increased intrathoracic pressures reduce deoxygenated blood returning into the thorax, thereby reducing the cardiac chambers volumes. To support this notion, the recent CLAIM study by Hohlfeld et al. showed that the reduced volume can be reversed by means of combined long-acting bronchodilators, causing deflation of the lung and increased end-diastolic filling of both the right and left ventricle and a significant increase in stroke volume (48). Moreover, up to 30% of COPD patients suffer from systolic or diastolic heart failure thereby enhancing in both pulmonary arterial wedge pressure and mPAP due to lung hyperinflation (49). Further, COPD patients do not show an increased hypertrophy of the left ventricle, but dysfunction. There is evidence that the systemic inflammation occurring in these patients might have a causal role in the pathogenesis of atherosclerosis (50). The high prevalence of left ventricular systolic dysfunction in individuals with COPD can be explained by the acceleration of the progression of coronary atherosclerosis by systemic inflammation, which leads to the development of ischemic heart disease. The high incidence of motor disorders of the left ventricle wall observed in patients with COPD and left ventricular dysfunction could also justify the relationship between both chronic processes.

Further, another explanation may be the existence of predominant COPD subphenotypes. Interestingly, Burrows et al. found that COPD patients with emphysema were less likely to demonstrate RV hypertrophy than other COPD patients, under the same pulmonary vascular resistance (51). Moreover, patients dying with emphysema did not exhibit RV hypertrophy, which was more common in COPD patients with chronic bronchitis (52). Further, Kawut et al. reported that cardiac complications are linked to more prominent airways disease and less parenchymal destruction, supporting a stronger link between the “chronic bronchitis” subphenotype than the “emphysema” subphenotype (53). The mechanism of reduced RV filling in emphysematous COPD may relate to several factors. As Watz et al. suggested that pulmonary hyperinflation reduces right atrial and RV filling in moderate-severe COPD (54), and lung volume reduction surgery for very severe COPD (which decreases hyperinflation) is associated with increased oxygen pulse (55). Long-term oxygen therapy (LTOT) can significantly improve the survival of hypoxemic COPD patients who also suffer from PH. Accordingly, the prognosis for PH will improve with LTOT therapy. In fact, LTOT is the only recommended therapeutic intervention to increase the survival rate of COPD patients with chronic hypoxemia. Approximately 20% of patients with COPD are prescribed LTOT (56). LTOT (13 h/per day) was prescribed for patients with COPD during the very early phase of the condition. This therapy was observed to be successful in patients with resting hypoxemia, however in nocturnal or exercise-induced hypoxemia LTOT exhibited no significant relief (56–58). Cor pulmonale also contributes to the mortality associated with COPD. Treatment in such cases involves LTOT administration for more than 16 h per day or the use of vasodilator drugs (59).

## Vascular Remodeling During PH

Vascular alterations have been shown to play an important role in the development of emphysema in both animal models and in patients with COPD. In patients with end-stage COPD, such remodeling is characterized by thickened walls or vascular occlusion, reducing the vascular lumen resulting in increased resistance and intravascular pressure. In PH-associated COPD, pulmonary ventricles and arteries undergo structural modifications (60). The most prominent feature of the vascular remodeling of blood vessels is the varying degree of thickening of the intimal and/or medial layer of muscular vessel layers in distal pre-capillary arterioles (distal muscularization) (61). Although smooth muscle cells (SMCs) are not resident intimal cells, studies in animal models have shown that SMCs can migrate from the media and proliferate in the intima following endothelial injury (61). It has become evident that intimal hyperplasia can be detected during early-stage COPD, resulting from the proliferation and migration of SMCs and associated with elastic and collagen fiber deposition (62). Immunohistochemistry analysis of ECM proteins from lung specimens of patients with COPD has shown that abundant elastin can be detected during the early stages of COPD and that the abundance of collagen is correlated with the degree of intimal thickness, suggesting that collagen deposition has important consequences for pulmonary vascular remodeling associated with COPD (60, 63–65). Sekhon et al. showed that, in rats, CS triggered proliferation of polymorphonuclear leucocytes (66). The effect of muscularization is prominent in small arteries (diameter <500 μm) (67–69). Immunohistochemistry, using SMC markers, of lung specimens from patients with PH-associated COPD showed positive expression of vimentin (indicating the expression of mesenchymal cells) and negative staining for desmin (an intermediate filament protein characteristic of cells of myogenic origin), indicating that less-differentiated SMCs contribute to an ongoing process of vascular remodeling (13, 67). Although the detailed molecular processes have not been identified, the occurrence of SMCs might be explained by the infiltration and differentiation of circulating bone marrow-derived progenitor cells, differentiation from resident precursor cells, the dedifferentiation of mature SMCs from the media that migrate to the intima (70), or transdifferentiation of endothelial cells to SMCs by endothelial-to-mesenchymal-transition. In this regard, bone marrow-derived progenitor cells are suggested to contribute on the one hand to vascular repair *via* differentiation into endothelial cells and on the other to vessel remodeling through differentiation into SMCs (71–73). Chronic hypoxia at high altitudes can also cause PH, but this condition is reversible upon returning to sea level. Together, these findings provide evidence of the primary reason for medial hypertrophy. By contrast, the remodeling of all vessel layers cannot be reversed by supplemental oxygen, either in cases of acute (74) or chronic COPD (75).

## Mechanisms of Vascular Remodeling During PH

The muscularization of vessels in the pulmonary region is a response to oxidative stress and endothelial cell (EC) injury. The integrity of ECs is lost due to apoptosis, following the accumulation of fluids and immune cells in the perivascular region. As a result of the immune response, bone marrow-derived precursor cells are recruited to the site, leading to the transition from ECs to mesenchymal cells (76, 77). Internal hypoxia has been proposed to be the primary mechanism underlying PH-associated COPD. Hypoxemia promotes vascular constriction *via* recruitment of immune cells and results in muscularization of arterioles. This remodeling affects the intima, media, and adventitia of vessels in the lung. Various studies have shown that pulmonary vascular remodeling and endothelial dysfunction occurs in animal models of lung emphysema (78), in patients with mild COPD not suffering from hypoxemia, and in smokers with normal lung function (72, 79). Presumably, oxygen therapy is unable to reverse PH in many patients with COPD. Nevertheless, many studies have shown that hypoxia plays a role in COPD, at least in severe forms of the disease.

## Mechanisms Leading to Vascular Remodeling During Hypoxia

PH occurs because of increased pulmonary vascular resistance (PVR) during chronic respiratory diseases. Multiple factors contribute to the increase in PVR (41, 80), but hypoxia during COPD is thought to be the major cause (41, 81, 82). Acute hypoxia causes pulmonary vasoconstriction, while chronic hypoxia induces structural vascular changes (remodeling) over time. During acute hypoxia, increases in PVR and mPAP are features of hypoxic pulmonary vasoconstriction. Chronic alveolar hypoxia causes morphological changes in the pulmonary vascular bed (remodeling) that are comparable to those seen in COPD patients with PH (including muscularization of pulmonary arterioles and thickening of the intima in muscular pulmonary arteries and arterioles).

Generalized hypoxia due to partial pressure of oxygen has both systemic and organ-specific effects (83–85). This type of hypoxia induces pulmonary vasoconstriction, peripheral vasodilation, and activation of a sympathetic-adrenergic stress response to increase cardiac output (86), while erythropoietin-stimulated red cell production is activated in the bone marrow. Hypoxic pulmonary vasoconstriction here represents an adaptive response in local blood perfusion to the alveolar ventilation situation, to prevent hypoxemia.

Hypoxia-dependent vasoconstriction is mediated by hypoxia-inducible transcription factor 1 alpha (HIF-1α), which promotes the activation of innate immune responses and inflammation in arterioles. Other transcription factors, such as forkhead box O (FoxO), CBF1/RBP-Jκ (recombination signal-binding protein for immunoglobulin kappa J region), peroxisome proliferator-activated receptor gamma (PPAR-γ), Krüppel-like factor 4 (KLF4), transcriptional coactivator pyruvate kinase isozyme PKM2, the corepressor CtBP1 [a member of the C-terminal binding protein (CtBP) family], and the Twist family bHLH transcription factor 1 (*TWIST1*) (87), were found to play a crucial role in PH and dysfunction of the right ventricle. However, of these transcription factors, HIF is most strongly implicated in PH pathogenesis, as it was observed that conditional deletions of HIF isoforms in mice improved vascular remodeling and augmented pulmonary arterial pressure post-chronic hypoxia (88). In alveolar macrophages, hypoxemia induces the expression of FIZZ1, also known as hypoxia-induced mitogenic factor (HIMF), thereby promoting smooth muscle contraction in pulmonary arterioles *via* interleukin-linked kinase 4 signaling mechanisms (76, 89). Patients with COPD who were regular smokers exhibited higher expression of HIF-1α, VEGF (a potent regulator of vascular permeability), and VEGF receptors (90). This in turn can activate the proinflammatory transcription factor nuclear factor-kappaB (NF-κB) (91, 92).

NF-κB modulates the expression of cytokine expression and thereby manipulates proliferative homeostasis in immune cells. Mechanistic insights into the hypoxia-induced expression of HIF and NF-κB suggest that both factors are activated in an IKK–transforming growth factor β-activated kinase 1 (TAK1)-dependent manner (93, 94). The hypoxic microenvironment inside the lungs also releases chemotactic factors, such as leukotriene B4 (LTB4), VEGF, and FIZZ1, which tend to increase hypoxia in the bone marrow as there is active mobilization of bone marrow-derived cells (such as mast cells, mesenchymal precursor cells megakaryocytes, and dendritic cells) to the lungs. Hypoxia-dependent transcription of NF-κB leads to transcription of phospholipase A_2_ (PLA_2_). Transcriptional activation of PLA_2_ results in activation of 5-lipoxygenase and chemotactic factor LTB4, eventually leading to the deposition of bone marrow-derived precursor cells (85).

**Figure 1:**
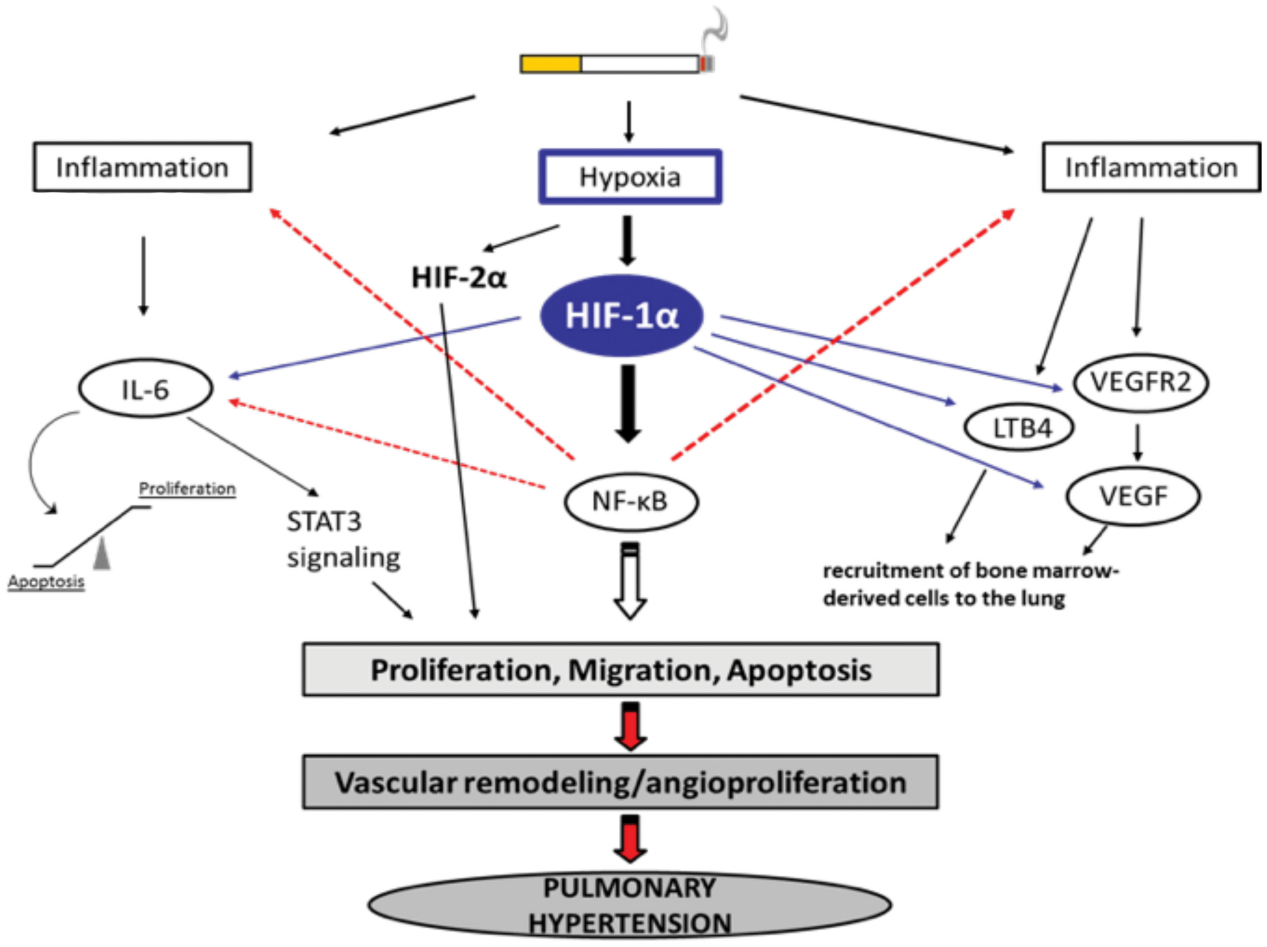
Selection of possible hypoxia-dependent mechanisms contributing to pulmonary vascular remodeling in COPD-PH. Cigarette smoke-induced airway obstruction and emphysema can result in hypoxia. Hypoxia, amongst others, activates HIF-1α which can trigger pathways associated with inflammation, the recruitment of bone marrow-derived cells, and alterations in proliferation/apoptosis balance of vascular endothelial and smooth muscle cells (SMC). Increased proliferation of SMCs causes narrowing of the vessels resulting in pulmonary hypertension. All important acronyms of the molecules are explained in the text.

IL-6 has received considerable interest as a mediator of COPD progression. IL-6 levels in serum from patients with COPD were found to be significantly elevated in comparison with healthy individuals (95). During hypoxia, IL-6-deficient mice showed less inflammation and a marginal reduction in pulmonary hypertension (96). IL-6 contributes to the increased migration of pulmonary artery smooth muscle cells (PASMCs) in chronic hypoxia-exposed vessels of the lung that are non-muscularized (96). IL-6 is upregulated following chronic hypoxia in mouse lungs, but it does not seem to be essential for the development of chronic hypoxia-induced PH. Chronic hypoxia with excessive IL-6 seems to change the mode of vascular remodeling toward angioproliferation (85). It has been reported that overexpression of IL-6 results in a significant decrease in the expression of lung protective protein bone morphogenetic protein receptor type 2 (BMRP2), through a signal transducer and activator of transcription 3 (STAT3)-microRNA cluster 17/92 pathway. The decrease in BMRP2 is driven by the modulation of the STAT3 pathway. BMPR2 is a member of the transforming growth factor-β (TGF-β) superfamily of growth factor receptors, which is involved in signaling pathways including protein kinase B/phosphatidylinosital 3-kinase (Akt/PI3K), phosphorylation of extracellular-signal regulated kinase (pERK), phosphorylation of c-Jun N-terminal kinase (JNK), phosphorylation of Smad1, and phosphorylation of phospho-mitogen activated protein kinase (p-p38MAPK) (97–99). Conversely, the effects of IL-6 overexpression have been found to be insufficient to cause pulmonary occlusions and aid the mobilization of bone marrow cells to vessels in the lung (85). It has been shown that mutations in the BMPR2 gene are linked to the development of PH (100) and that BMPR2 expression is reduced in the pulmonary vasculature in patients with PH (101). On the other hand, it is assumed that IL-6 affects the balance between apoptosis and proliferation of PASMCs and pulmonary artery endothelial cells (PAECs), leading to vascular remodeling (102). Therefore, overexpression of IL-6 induces the angioproliferative growth factor VEGF and intracellular ERK, resulting in increased proliferation. In parallel, IL-6 expression is followed by a downregulation of TGF-β and proapoptotic MAP kinases (JNK1, p38^MAPK^) (103) and upregulation of B-cell lymphoma 2 (Bcl2), an inhibitor of apoptosis. Accordingly, IL-6 can trigger vascular remodeling by inducing signaling pathways that lead to increased proliferation and decreased apoptosis of PASMCs and PAECs (Figure 1). There are some reports about the role played by BMPR1αin pulmonary arterial hypertension (PAH). In a mouse model, deletion of BMPR1α did not significantly modify the dynamics of blood flow in the distal vasculature of the lung as a response to hypoxia, however the hemodynamics of proximal pulmonary arteries were changed; a deficiency in BMPR1α further decreased excessive dilation, as a result of collagen accumulation (104). Arterial stiffness in turn altered the function of the right ventricle.

In addition to the modulation of signaling cascades, IL-6 also modulates the homeostasis between pro- and anti-apoptotic proteins, thereby encouraging vascular remodeling. IL-6 overexpression upregulates Bcl2 and survivin, inhibitors of apoptosis, leading to the inhibition of apoptosis. IL-6 promotes cell proliferation *via* activation of the VEGF and MAPK pathways (85). This cytokine also participates in cell differentiation and proliferation of the ECM, thereby causing hyperplasia in airway goblet cells and squamous metaplasia in small airway cells. The proliferation of ECM activates the ERK and p38 signaling pathways by increasing the expression of type I/III collagens, laminin, and fibronectin and decreasing the expression of proteoglycans and elastin (94).

**Figure 2:**
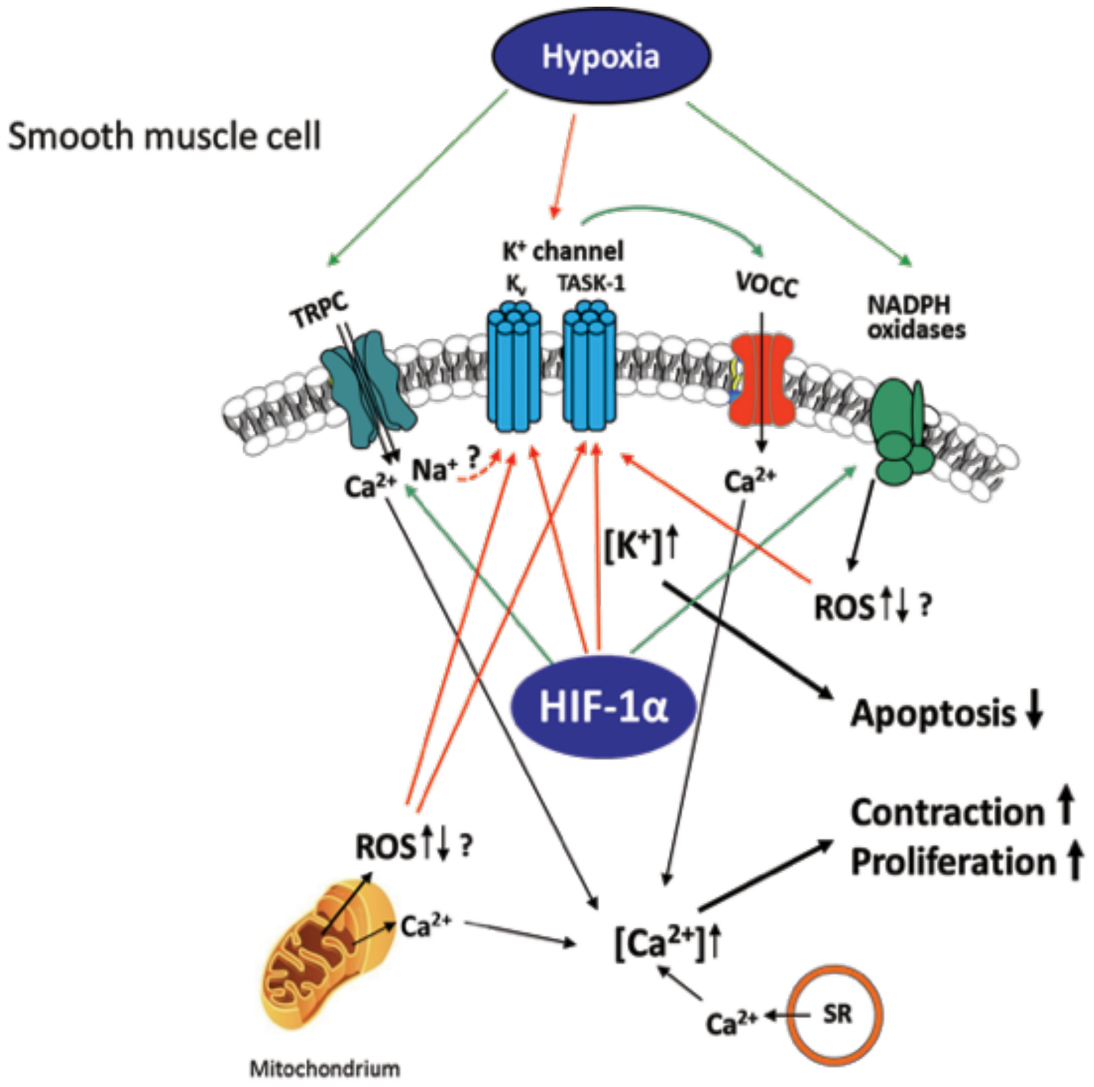
Hypoxia-induced ion channel-mediated increase in proliferation, contraction, and decrease of apoptosis of SMCs contributing to pulmonary vascular remodeling. K_v_ and TASK-1 channels are downregulated and less active after chronic hypoxia leading to accumulation of K^+^ within the cell (mediating apoptosis-resistance) and membrane potential depolarization of the SMCs. This depolarization causes opening of voltage-operated Ca^2+^ channels (VOCC), especially L-type channels, which mediate Ca^2+^ entry. Hypoxia-dependent ROS regulation derived from NADPH oxidases and/or mitochondria is suggested to inhibit the K^+^ channels, although it is unclear whether an increase or decrease of ROS occurs in hypoxia. Transient receptor potential channels (TRPC)-mediated Ca^2+^ or Na^+^ influx (speculatively by gating K^+^ channels) was also shown to be essential for the intracellular Ca^2+^ increase in at least acute hypoxia. Ca^2+^ release from mitochondria and sarcoplasmic reticulum (SR) was shown to additionally increase Ca^2+^ within the cell. Thus, mediated contraction and proliferation of the SMCs can contribute to vascular remodeling. Colored arrows depict either activation (green) or inhibition (red).

Hypoxemia indirectly modulates the expression of angiotensin II, serotonin, platelet-derived growth factor, and metalloproteinases present on the walls of arteries in the lungs, leading to alterations in vascular cross-sectional areas and pulmonary hypertension. The pathways may be interrelated with intracellular ion concentrations. It has been previously reported that potassium channels play a crucial role in inducing excitation in smooth muscle. Inhibition or activation of vascular SMCs can therefore cause changes in their membrane potential, leading to augmentation of calcium ion concentrations inside the cells and propagating vasoconstriction (Figure 2). It has been shown that, under chronic hypoxic conditions, voltage-gated K^+^ channel (Kv) currents are decreased (105–107), which is most likely mediated by ROS derived from mitochondria (108–112) and/or NADPH oxidases, such as NOX4 (113). Furthermore, influx of calcium ions *via* transient receptor potential ion channels (TRP) has also been associated SMC proliferation and hypoxia induced vasoconstrictions in the lungs (114). Under hypoxia, the membrane potential is depolarized by between 15 and 20 mV and arteries are constricted to about 300 μM diameter. The generation of ROS in mitochondria *via* NADPH oxidase has a considerable effect on potassium ion conductance and membrane potential (115, 116). The voltage-independent, two-pore domain K^+^ channel, TWIK-related acid-sensitive K^+^channel, (TASK)-1, was shown to be inhibited by hypoxia, leading to membrane depolarization and calcium ion entry through L-type channels. Mutations in the potassium channel subfamily K member 3 (*KCNK3)* gene, which encodes for TASK-1, have been reported in patients with PH. To date, six different mutations in *KCNK3* have been studied in patients diagnosed with PH (117–119). Another protein, the 30-kDa four and a half LIM domain protein 1 (FHL1), has been shown to participate in the induction of hypoxia-induced migration as well as in proliferation of PASMCs thus indicating its importance in vascular remodeling during PH-associated COPD and in patients with idiopathic PAH. The increase in FHL-1 causes migration of PASMCs and their elevated proliferation, contributing to vascular remodeling (120). However, the precise molecular mechanisms underlying this remain unclear (120–122).

## Mechanisms Independent of Hypoxia that Cause COPD and PH

It has long been suggested that hypoxia is the primary driving force behind the development of PH in COPD. This was supported by studies that showed a close relationship between mPAP and/or pulmonary resistance and alveolar hypoxia (123, 124). However, there is also evidence for other causal factors, independent of hypoxia. First, it has been demonstrated that oxygen therapy is unable to completely reverse PH in COPD (75, 125). Second, analysis of pulmonary vessels from COPD patients with PH demonstrated prominent intimal thickening, medial hypertrophy, and muscularization of small arterioles (126). In contrast, hypoxia-induced vascular remodeling has been shown to be restricted to the media. In addition, these pulmonary vascular alterations occurred in non-hypoxic patients with mild airflow obstruction and smokers without any parenchymal disorder, suggesting that vascular remodeling may be driven by mechanisms independent of hypoxia/hypoxemia (79). Consistent with these findings in humans, studies of mice exposed to tobacco smoke demonstrated that pulmonary vascular remodeling and PH preceded the development of emphysema and was independent of hypoxia (27, 29). Furthermore, these studies showed that gene expression patterns linked with pathways associated with PH and COPD, such as apoptosis, proliferation, oxidative stress, ECM, and inflammation, were completely different compared with gene expression patterns during chronic hypoxia-induced vascular remodeling. Interestingly, the combination of CS and hypoxia act synergistically to affect the vasculature. Experiments with guinea pigs exposed to CS and to hypoxia showed increased mPAP and more pronounced remodeling in small vessels compared with guinea pigs exposed to a single stimulus (28).

Animal studies have shown that CS has a direct effect on the parenchyma and the vasculature. CS has also been shown to increase the expression of genes that encode vasoactive mediators in pulmonary arteries (127, 128). Guinea pigs exposed to chronic CS developed emphysema that was associated with reduced lung capillary density (129). Additionally, cigarette smoke extract (CSE) can induce endothelin-1 (ET-1) in PAECs (130) and reduce prostacyclin synthase expression (131). Furthermore, CSE induces the production of superoxide in ECs, promoting peroxynitrite formation (132). This strong oxidant radical has been shown to suppress VEGFR2 expression, followed by a reduction in EC maintenance and growth. It has been suggested that the cGMP pathway is downregulated following exposure to CS. Congruently, CSE-induced EC apoptosis *via* p53 (133) can be prevented by the PDE5 inhibitor sildenafil (134), followed by an increase in cGMP levels.

The inability of LTOT to completely reverse the vasoconstriction and remodeling seen in patients with PH-associated COPD indicates that hypoxia-independent factors are also involved in the development of PH in COPD patients. This is supported by finding that pulmonary vascular alterations were also observed in non-hypoxic patients with mild airflow obstruction and smokers with no parenchymal disorders. CS-exposed C57BL/6 mice developed emphysema. In addition, the incursion of large numbers of neutrophils and macrophages and activation of NF-κB and inflammatory cytokines were observed. Further studies have reported that smoke-induced PH and emphysema in mice can be reduced by inhibiting the expression of inducible nitric oxide synthase (iNOS) and activating the expression of soluble guanylate cyclase (sGC) (29). Furthermore, these authors showed that the inhibition of iNOS regulates genes that support lung regeneration through the formation of new alveoli (neoalveolarization) (29, 135). Studies with L-NIL (N6-(1-iminoethyl)-L-lysine dihydrochloride), an iNOS inhibitor, showed repression of matrix metallopeptidase 9 (MMP9) *via* amplified transcription of expression of metalloproteinase inhibitor 3, TIMP3, resulting in decreased parenchymal destruction. It was also reported that L-NIL treatment supported the formation of elastic fibers in the lungs, thereby inducing active pulmonary repair. In addition, the repression of pro-proliferative (*Fgf10 and Ccna1*) and apoptosis-inducing factors and reduced proliferation of granulocytes, macrophages, and activated T cells post iNOS inhibition was found to have a positive effect on vessels and neoalveolarization, in support of vascular regeneration (29). CS increases EC permeability *via* the activation of Ras homolog family member A (RhoA) and myosin light chain (MLC) kinase. Further, CS promotes the release of vasoconstrictors and pro-mitogenic markers, such as ET-1 and thromboxane A2, which eventually results in the remodeling of vessels and pulmonary cell dysfunction (27, 130, 134, 136). Prostacyclin, a well-known vasodilator present in ECs and SMCs, is known to be inhibited in smoke-induced PH. A loss of prostacyclin in the lungs was found to result in increased platelet adhesion and endothelial dysfunction in PH-associated COPD patients (137).

## Endothelial Dysfunction (ED) in the Pathogenesis Of PH in COPD

**Figure 3:**
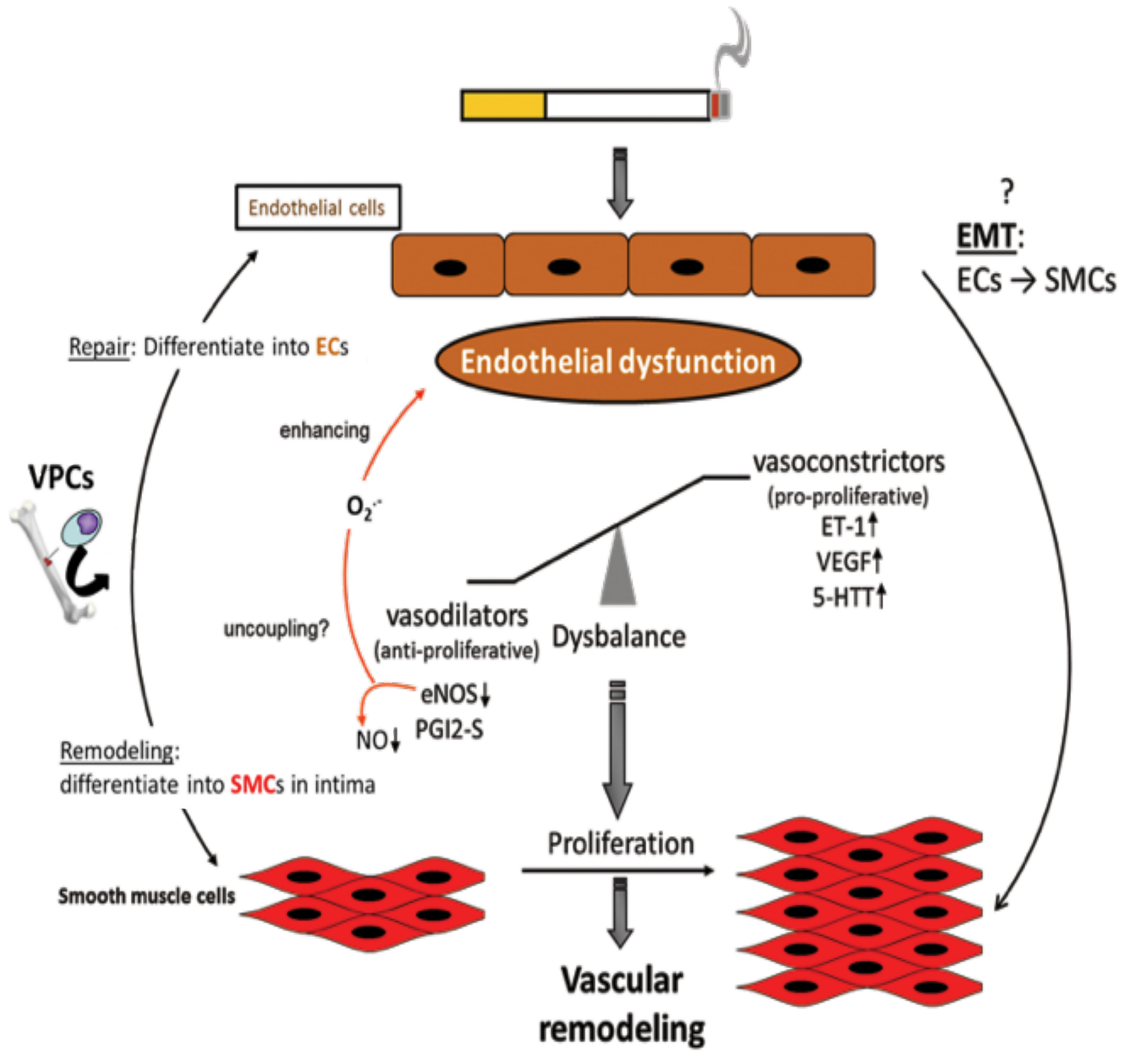
Endothelial dysfunction as causing factor for development of vascular remodeling. Cigarette smoke and inflammatory mediators can cause endothelial dysfunction which is triggered by a disbalance of vasodilative and vasoconstrictive molecules towards an excess of vasoconstrictors and damage/dysregulation of EC signaling. Additionally, vascular progenitor cells (VPCs) are attracted to the damaged endothelium. Such VPCs can either contribute to repair by differentiation into ECs or to remodeling by differentiation into SMCs. Furthermore, an endothelial-to-mesenchymal-transition (EMT) may occur resulting in an SMC phenotype. Vasoconstriction and altered endothelial cell signaling are stimuli for SMCs to proliferate resulting in vascular remodeling, increased pulmonary artery pressure, and finally in pulmonary hypertension.

The pathogenesis of PH in COPD is thought to be driven by an endothelium-derived vasoconstrictor/dilator imbalance caused by ED. Thus, ED in the walls of arteries in the lungs contributes to the development of PH in patients with COPD. This dysfunction has been measured by analyzing the nitric oxide-dependent relaxation of arterial rings in the lungs in response to dose-dependent increases in exogenous acetylcholine and adenosine diphosphate (ADP) (78, 138, 139). In emphysematous lungs, the expression of VEGF and VEGFR was significantly reduced. The extent of pulmonary injury was further increased following exposure of the lungs to CS. The growth and proliferation of inadequately differentiated SMCs and deposition of ECM proteins in arterial walls in the lungs contributed to the dysfunction and progression of the disease. The process of ED is also associated with decreased expression or uncoupling of endothelial nitric oxide synthase (eNOS), which might contribute to the development of PH (Figure 3). In contrast, the expression of VEGF and serotonin transporters seems to be increased (140, 141). ECs are known to be important for the regulation of vascular homeostasis. They control the vascular tone and affect pulmonary vessel adaptations to changes in blood flow and in response to hypoxia (142–145). Dysfunction of the endothelium has been reported in patients with end-stage COPD after lung transplantation (140), as well as in patients with mild-to-moderate COPD (146). In general, endothelial function is affected by the expression of various vasoreactive substances that control vasoconstriction (and are also pro-proliferative for SMCs) or vasodilation (and are also anti-proliferative for SMCs). In patients with primary or secondary PH and COPD, the protein ET-1 was shown to increase (147). Vasodilative mediators, such as eNOS (147, 148) and prostacyclin synthase (PGI_2_-S), were downregulated in pulmonary arteries (131). CSE can decrease the expression of PGI_2_-S in human PAECs (131), suggesting that its downregulation is a direct effect from the ingredients in CSE. After exposure to CS for 8 months, the downregulation of eNOS in lungs and vessels was observed, concomitant with the development of emphysema and PH (29). In addition, eNOS-deficient mice developed emphysema and PH following exposure to CS, whereas iNOS-deficient mice did not (29). Other changes in the presence of CS include increased injuries to ECs, increased infiltration of neutrophils, increased lipid peroxidation, excessive oxidative stress, and imbalances between the expression of vasoconstrictors and modulators (78) (Figure 3).

## Altered Profiles of Inflammatory Cells

Acute exacerbation due to increased inflammation in the lungs is the most important characteristic identified and studied in patients with COPD. During progression of the disease, excessive mucus, containing inflammatory cells, accumulates in the blood vessels thereby increasing the tissue volume in the bronchial wall. The increase in tissue volume can be measured by the infiltration of both innate and adaptive inflammatory markers, such as neutrophils, macrophages, and CD4 and CD8 lymphocytes. Neutrophils contribute significantly to the production of ROS, cytokines, and chemokines during lung inflammation. The inflammation is triggered *via* the activation of Toll-like receptors, followed by transcriptional activation of NF-κB and activation of STAT pathways. During pulmonary inflammation, patients with COPD exhibit higher expression of inflammatory cytokines, such as IFN-γ and TNF-α, along with IL-1, IL-6, and IL-8. Reports have also indicated that IL-17 plays a role in COPD progression and inflammation (149–152). In patients with COPD, an increased number of inflammatory cells invade the adventitia of pulmonary muscular arteries. These cells are predominantly activated CD8^+^ T-cells (140, 153). Numbers of these lymphocytes are also increased in the arterial adventitia of smokers with normal lung function. The ratio of CD4^+^/CD8^+^T-cells is reduced compared with the ratio in non-smokers and is comparable to the situation in patients with mild-to-moderate COPD (153). In addition, an association between IL-6 expression and elevations in mPAP supports a role for inflammation in the pathogenesis of PH (in COPD) (154). This is particularly relevant, because the vascular adventitia has been shown to harbor inflammatory cell progenitors, e.g., CD45^+^ macrophage progenitors, independent from bone marrow-derived monocytes (155–157). Upon activation, these progenitors deliver F4/80^+^ macrophages that serve as a local source for high levels of VEGF production (157). The potential contribution pulmonary vessel adventitia-derived inflammatory cells, e.g., macrophages, make to PH and the proliferation of vascular adventitia-resident SMC progenitors has yet to be studied.

## The Effect of Oxidative and Nitrosative Stress on Vascular Physiology

Increased production of ROS in the endothelium is an important characteristic of pulmonary endothelium dysfunction in PH. During oxidative stress in patients with PH, the transcription factor nuclear factor erythroid 2-related factor 2 (Nrf2) fails to activate the expression of antioxidant enzymes, such as superoxide dismutase (SOD) and catalase, thereby increasing the level of ROS in the body. Also, increased production of superoxide radicals reduces the availability of NO, as superoxide ions merge with NO to form peroxynitrite, which in turn increases the expression of inflammatory markers and leukocyte infiltration *via* the expression of adhesion molecules in the endothelium. The formation of peroxynitrite further contributes in depolarizing potassium ion channels and increasing calcium ion concentration inside cells, eventually leading to tissue injury due to increased vascular permeability. Superoxide ions can generate downstream toxic products, including hydrogen peroxide (H_2_O_2_), which in turn activates the phosphorylation of NF-kB and leads to the activation of inflammatory responses. Activation of inflammasomes due to oxidative stress further perpetuates inflammation in the body (151, 158). The elevated production of superoxide and H_2_O_2_, together with reduced NO bioavailability, make a fundamental contribution to vascular remodeling and the development of emphysema. Elevated concentrations of H_2_O_2_ and 8-isoprostane, both of which are oxidative stress markers, are found in the exhaled breath condensate of smokers and ex-smokers, as well as during exacerbations (159, 160). ROS have been shown to negatively affect the function of antiproteases, such as α1-antitrypsin and secretory leukocyte proteinase inhibitor (SLPI) (16, 161). Consequently, a protease/anti-protease imbalance accelerates the degradation of elastin in the lung parenchyma, resulting in emphysema. Angiotensin II is involved in NADPH oxidase-generated superoxide production, mediated by angiotensin type I receptor, which is converted to H_2_O_2_, with SOD acting as a second messenger. This pathway induces hypertrophy or hyperplasia in vascular SMCs (162, 163). This angiotensin II-induced process has been shown to be inhibited by the flavoprotein inhibitor DPI (162), catalase, and knockdown of p22^phox^, which supports the involvement of NADPH oxidases in the vasculature (164–166).

ROS promote vascular remodeling by increasing the deposition of ECM proteins. In particular, collagen and elastic fibers are degraded by proteinases, specifically MMPs. MMPs are secreted, in an inactive form, by macrophages and vascular SMCs (167). ROS, such as peroxynitrite, have been shown to activate MMP-2 and -9 in human SMCs, followed by degradation of the basement membrane and elastin (168, 169). A hypertension model involving aldosterone-induced systemic oxidative stress revealed that endothelin-1-associated processes are the main contributors to vascular remodeling (170, 171). Similarly, redox-sensitive inflammatory processes are known to induce vascular remodeling. In particular, increased expression of the inflammation-related intracellular adhesion molecule-1 (ICAM-1) has been shown in the aorta of aldosterone-treated rats (170, 171). Furthermore, angiotensin II-induced oxidative stress results in tissue hypertrophy associated with an increase in ICAM-1 expression. Macroscopic and microscopic examinations of COPD emphysematous lungs using hematoxylin and eosin staining showed that the alveolar septa were extremely thin and avascular (172). This indicates that pulmonary endothelial dysfunction might be the key element in COPD pathogenesis.

## Endothelial Dysfunction in COPD in Cardiovascular Diseases (CVDs)

**Figure 4:**
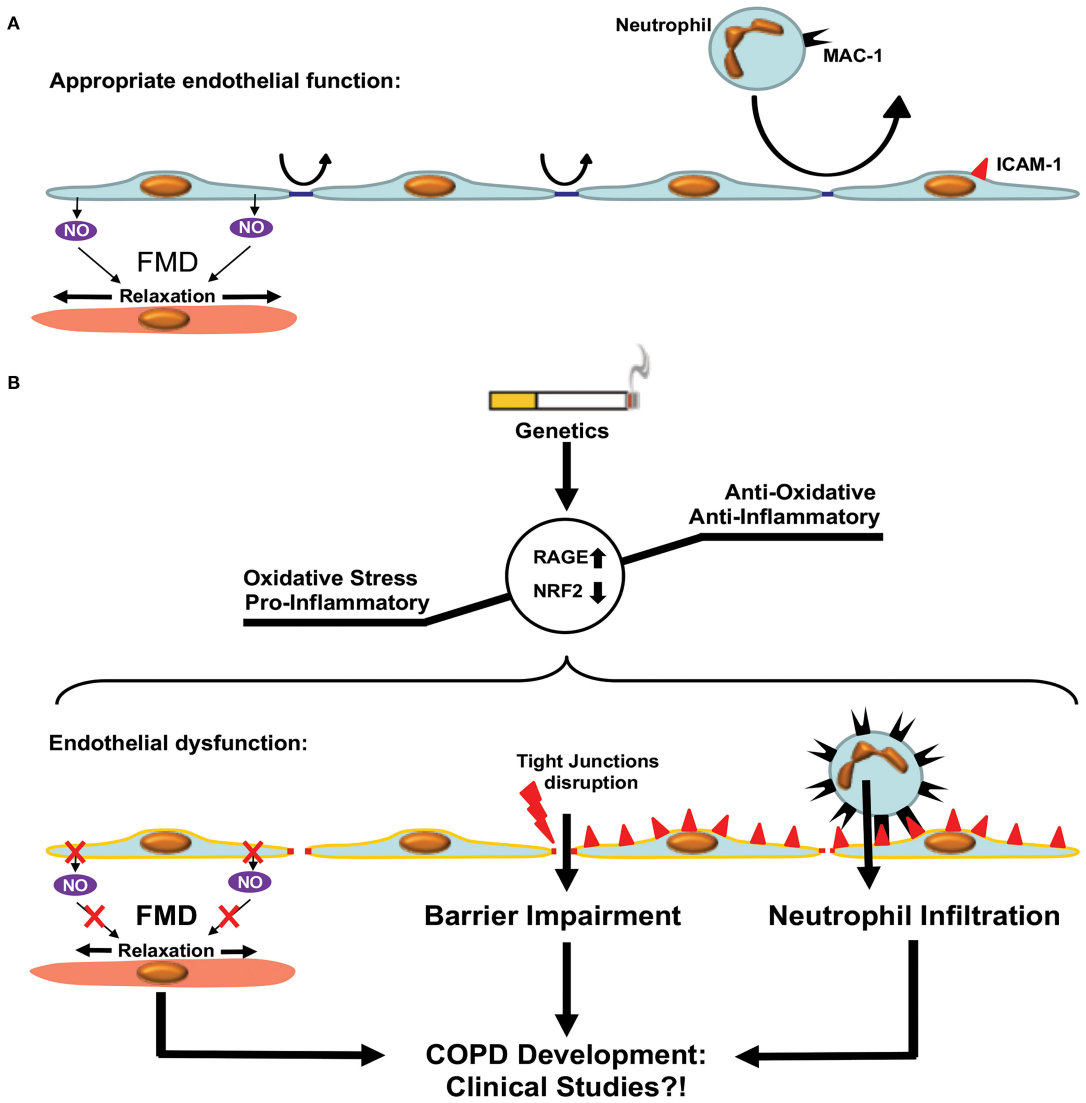
Endothelial dysfunction in COPD. **(A)** Appropriate endothelial function under physiological conditions. The endothelium shows adequate flow mediated dilation (FMD) via relaxation of smooth muscle cells within the vascular media. Paracellular permeability is limited by intact tight junctions. Low adhesion molecule expression limits neutrophil extravasation in pulmonary tissue. **(B)** Development of COPD is accompanied by enhanced oxidative stress and a pro-inflammatory state resulting in a dysfunctional endothelium. Dysfunction is characterized by reduced FMD, impaired endothelial barrier function due to disruption of tight junctions and enhanced expression of adhesion molecules facilitating neutrophil extravasation. These processes promote COPD development via increased inflammation. Clinical studies are required in order to test the beneficial effects of therapies targeting ED in COPD patients.

The endothelium forms a continuous monolayer and thereby a regulated barrier that separates the intravascular blood compartment from surrounding tissues (173, 174). ED is classically defined as impaired NO-mediated vascular relaxation (Figure 4). In a broader sense, ED encompasses a state in which ECs are activated, additionally characterized by endothelial barrier impairment and reduced anti-adhesive and antithrombotic properties (175).

Recent studies have shown that the microvascular barrier is impaired in patients with COPD and that the level of impairment is correlated with the severity of airway obstruction (176, 177). This could be attributed to the disruption of endothelial tight junctions observed in patients with COPD, even in the absence of CS (178, 179). Immune cells, especially neutrophils, are critically involved in the pathogenesis of COPD. This has been reviewed in details elsewhere (180, 181). Neutrophil accumulation was observed in the lungs of patients with COPD, in clinical settings, using radiolabeled neutrophils and single-photon emission computerized tomography (SPECT/CT) imaging (182). Neutrophil accumulation within the pulmonary tissue requires extravasation through the endothelial barrier, which is an active process involving adherence to ECs and migration either between (paracellular diapedesis) or through (transcellular diapedesis) ECs. Neutrophil MAC-1 (α_m_β_2_, CD11b/CD18, and complement receptor type 3) interactions with endothelial ICAM-1 promote endothelial transmigration of neutrophils. Neutrophil adhesion and migration across the endothelium were shown to be upregulated in patients with COPD, presumably *via* upregulation of MAC-1 expression in neutrophils (183). Accordingly, levels of ICAM-1 were shown to be elevated in patients with COPD (184). Furthermore, ICAM-1, as well as P-selectin, another endothelial adhesion molecule involved in neutrophil transmigration, was inversely associated with first forced expiratory volume (FEV1). Furthermore, enhanced soluble ICAM-1 levels are independently associated with emphysema progression in the general population (185).

In addition to the presence of ED in animal studies of COPD, clinical studies suggest a dysfunction of the endothelium even prior to the onset of COPD. This has been ascribed to excessive tobacco consumption, since normal lungs of smokers showed intimal thickening of small pulmonary arteries, similar to that seen in the lungs of COPD patients, when compared with lung tissues of non-smokers (146).

Flow-mediated dilation of the brachial artery analyzed by ultrasound, the reference method used to determine ED in humans, is reduced in early COPD and associated with FEV1 reduction and a higher percentage of emphysema in CT scans of former smokers (186–188). In addition to ED, direct injury of ECs seems to play a critical role in COPD, as pulmonary septa appear almost avascular (172) and CT scans of patients with COPD show vascular pruning of small arteries that can predict the clinical severity of disease and mortality (189, 190). This has been linked to impaired VEGF signaling, as VEGF and VEGFR2 expression are reduced in areas of the lungs with emphysema in patients with COPD (191), and VEGFR inhibition resulted in an emphysema phenotype in animal studies (192–194). In contrast, enhanced expression of HIF-1α, VEGF, and VEGFR was observed in human patients with COPD and reflected disease severity. This led to the assumption that VEGF signaling is increased in non-emphysema tissue in COPD patients, while it is decreased in emphysematous COPD parenchyma (90). It is important to mention that current knowledge on the vascular involvement in COPD/emphysema is based upon the pioneering work of Averill A. Liebow (172).

Patients with COPD have an increased risk of suffering with CVDs (50). This is of particular relevance, as ED is a common feature in both COPD and the development of atherosclerosis, which in turn can cause CVDs such as myocardial infarction and stroke (50, 175, 195). Interestingly, ED in patients with COPD exhibits an intermediate state between healthy patients and patients suffering from coronary artery disease (196). In a murine model of atherosclerosis (*Apo.E*^−/−^), increased oxidative stress was suggested to link ED with COPD pathogenesis, especially with regard to the development of emphysema (197). Several studies have shown increased vascular oxidative stress levels in COPD patients, which is associated with a reduction in FEV1 (198–200). The receptor for advanced glycation end products (RAGE) seems to play a major role in this process (Figure 4). Genetic deletion or pharmacologic inhibition of RAGE protects against the development of CS-induced emphysema (201). Furthermore, genome-wide studies of single nucleotide polymorphisms linked RAGE to the development of emphysema in COPD (202, 203). Therefore, targeting vascular oxidative stress-mediated ED seems to be a promising treatment for COPD. However, studies investigating anti-inflammatory therapy in patients with COPD were mainly conducted without determining the effect on vascular dysfunction (204–208). So far, only animal studies have provided evidence that anti-oxidative treatment of ED shows a beneficial effect on COPD, i.e., by the activation of the transcription factor Nrf2 (94, 207, 209). Therefore, investigating the targeting of ED in COPD, e.g., with anti-oxidative pharmaceuticals, with a concomitant analysis of effects on flow-mediated dilation, remains a task for the future.

## Role of the No–sGC–cGMP Axis in the Association Between COPD and Vascular Remodeling/PH

Nitric oxide has been suggested to play an important role in CS-induced emphysema and PH in mice (29). It has been shown that CS induces upregulation of iNOS; this was predominantly observed in small pulmonary vessels and associated with increased NO generation. Interestingly, iNOS was downregulated during the early phase of disease, in both mice and human patients with COPD. The vasodialative effect of NO was most likely abolished because of the abundance of ROS from both external (CS) and internal sources. It was also suggested that the formation of peroxynitrite had pro-apoptotic and anti-proliferative effects on alveolar epithelial cells type II (AECII) and ECs, resulting in the development of emphysema, vessel loss, vascular remodeling, and an increase in the level of nitrotyrosine. It is assumed that the decrease in eNOS level was associated with the uncoupling of this enzyme, followed by a switch from NO to superoxide production. Interestingly, in iNOS-deficient but not eNOS-deficient mice, vascular remodeling, PH, and emphysema did not occur. Additionally, treatment with an iNOS inhibitor (L-NIL) prevented the development of disease and promoted lung regeneration in mice exposed to CS.

**Figure 5:**
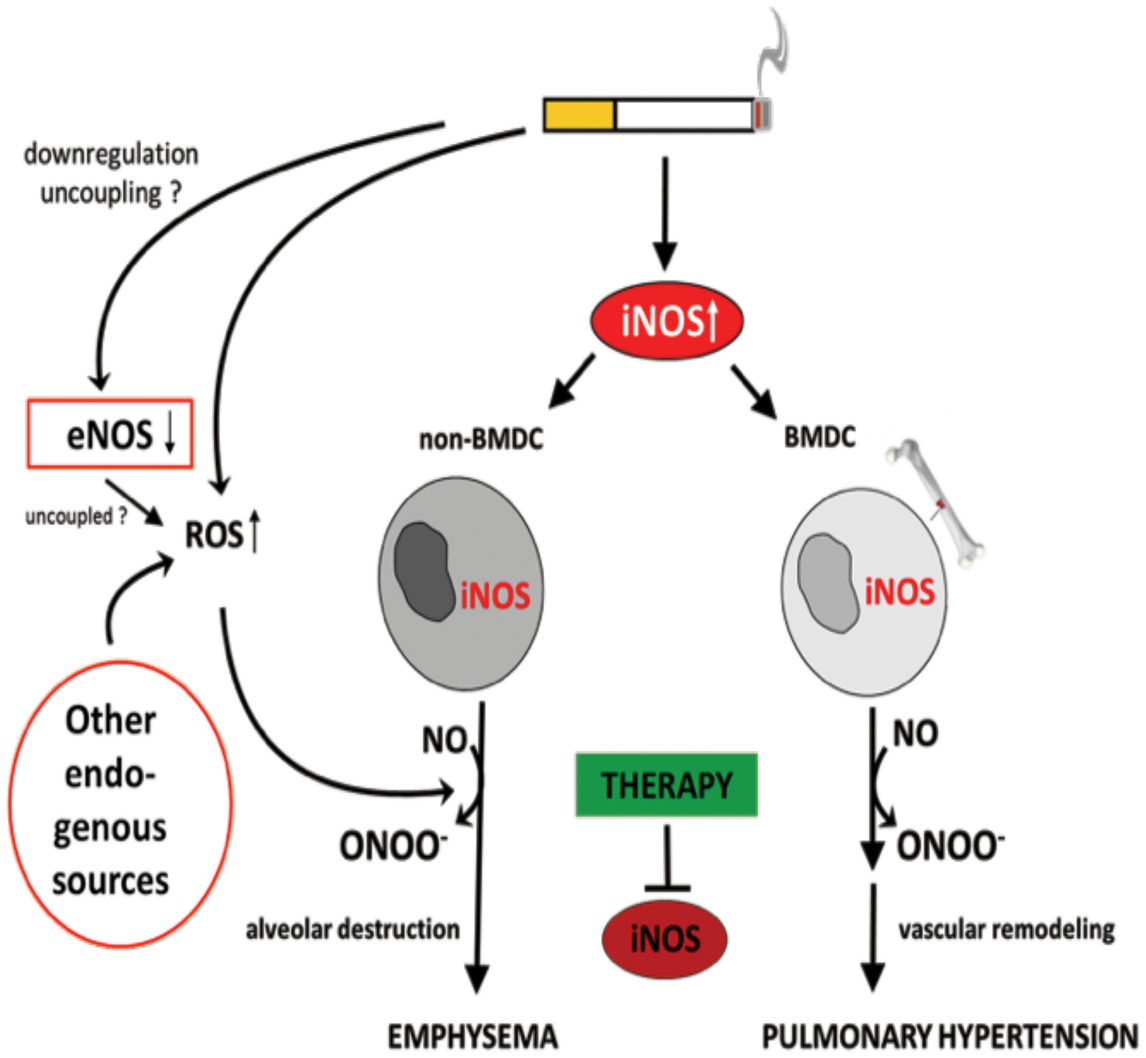
Identification of inducible NO synthase (iNOS) as an essential factor for the development of cigarette smoke-induced emphysema and pulmonary hypertension in mice. Cigarette smoke-mediated upregulation of iNOS leads to excessive NO production. The formation of peroxynitrite, resulting from the reaction of NO with superoxide, was suggested to mediate emphysema and PH development. Superoxide can derive from cigarette smoke itself and/or from uncoupled eNOS, NADPH oxidases, xanthine oxidases, cyclo- and lipooxidases, and mitochondria. Of interest, iNOS generated by non-bone marrow-derived cells (N-BMDC), possibly vascular cells, leading to lung destruction resulting in emphysema whereas elevated iNOS expression in bone marrow-derived cells (BMDC) causes vascular remodeling. Treatment with specific iNOS inhibitor L-NIL prevents or even reverses pathological alterations.

It has been demonstrated that iNOS-carrying bone marrow-derived cells mediate the development of PH, while emphysema is dependent on iNOS in non-bone marrow-derived cells (29) (Figure 5). These results clearly show that the pathophysiology of PH and emphysema is partly independent. Furthermore, this might also be a reason why not all patients with COPD suffer from PH. In animal studies, it was further demonstrated that the stimulation of sGC, an enzyme that uses (i) NOS-generated NO to produce cGMP from guanosine triphosphate (GTP), prevented the CS-induced development of vascular remodeling but also emphysema (210). cGMP acts as a second messenger mediating vasodilation. In addition to the vasodilatory effect, cGMP was shown to effect on proliferation, platelet aggregation and recruitment of inflammatory cells (211). These authors further showed that, riociguat (approved for PAH and chronic thromboembolic PH treatment) which promotes the NO-cGMP pathway, not only prevented tobacco smoke-driven PH development but also prevented airspace enlargement in smoke-exposed mice (210, 211). Similarly, in another recent study from Pichl et al. showed the riociguat treatment in the mouse model of smoke-induced PH and emphysema reversed fully established emphysema, muscularization of small pulmonary vessels, and RH hypertrophy and had beneficial effects on small cohort of COPD patients (212). Moreover, the same group also investigated another drug BAY 41-2272 which also stimulates sGC in tobacco smoke- exposed guinea pigs reduced vascular remodeling and prevented emphysema development (210). In, another study by Paul et al. also demonstrated by treating this drug BAY 41-2272 to guinea pigs that were chronically exposed to smoke exhibited similar effects with decreased extent of emphysema, RV hypertrophy, and improved pulmonary haemodynamics (213). These studies demonstrate the importance of sGC playing crucial role in the pathology of COPD. Therefore, other downstream molecules of this pathway were focused in preclinical studies. Namely, blocking of cGMP degradation by PDE5 inhibitors such as sildenafil (214) or tadalafil (27), prevented the development of PH in smoke-exposed guinea pigs and mice, respectively. Moreover, treatment with the PDE4 inhibitor demonstrated a significant protective effect on emphysema and PH development, suggesting that cAMP plays an important role in the pathology of COPD. These findings implicate an important role for the NO–sGC–cGMP axis in the physiology and pathophysiology of the pulmonary vasculature (29). The dysregulation of this system has previously been suggested to contribute to pulmonary diseases and PH (215–217). In line with previous findings (29), impairment in vascular remodeling was associated with the prevention of emphysema, although causality was not investigated in these studies.

## Role Of ROS in Vascular Remodeling

While it is known that CS leads to ROS-mediated oxidative stress, other sources of ROS that affect vascular remodeling have yet to be fully resolved. Various sources of ROS, such as mitochondria, NADPH oxidases, xanthine oxidase, cyclooxygenases, lipooxygenases, and uncoupled eNOS, must be considered (215, 218–221). Furthermore, especially in the lung, mitochondria have been implicated in vascular remodeling, and imbalances in mitochondrial ROS production seem to play an important a role in this process (222–226). Moreover, recent evidence suggests that mitochondrial ROS are causatively linked to the development of PH (227). However, the mechanisms of mitochondrial ROS production have not yet been fully elucidated. The vasculature in non-hypoxic PAH models has suggested a decrease in mitochondrial ROS, whereas a chronic hypoxia model of PH demonstrated increased mitochondrial ROS (222, 228, 229). According to some researchers, mechanistic discrepancies in mitochondrial ROS production might be based on “different experimental conditions, species differences, and perhaps complexities of how pure hypoxic stress may interface with other triggers of PH” (227). In addition to this phenomenon, significant alterations in mitochondrial metabolic pathways may drive a metabolic shift (“cancer theory of PH”) in PASMCs, triggering vascular remodeling and PH (227, 230, 231). In terms of COPD, recent data provide some evidence that mitochondrial ROS might play a role in lung remodeling/emphysema development. The overproduction of the alternative oxidase (AOX), which bypasses the cytochrome segment of the respiratory chain, attenuated CS-induced lung tissue destruction and loss of function in mice chronically exposed to CS for 9 months. This implicates mitochondrial respiratory inhibition as a key pathogenic mechanism of CS toxicity in the lung (232).

Regarding vascular remodeling, ROS are known to affect various intracellular signaling cascades, such as activation of ERK, MAPKs, protein tyrosine phosphatases, transcription factors such as NF-kB and AP-1, and receptor and non-receptor tyrosine kinases, which have been shown to be involved in cardiovascular remodeling and vascular damage. In addition, monocytes and lymphocytes are able to infiltrate cardiovascular tissues and pulmonary vessels, while inflammatory processes are often related to immune defense or CS. Previous studies have also shown that macrophages contribute to COPD development and that NADPH oxidase plays a crucial role in this regard (233, 234).

## Downregulation of Neprilys in Affects Pulmonary Vascular Remodeling

**Figure 6:**
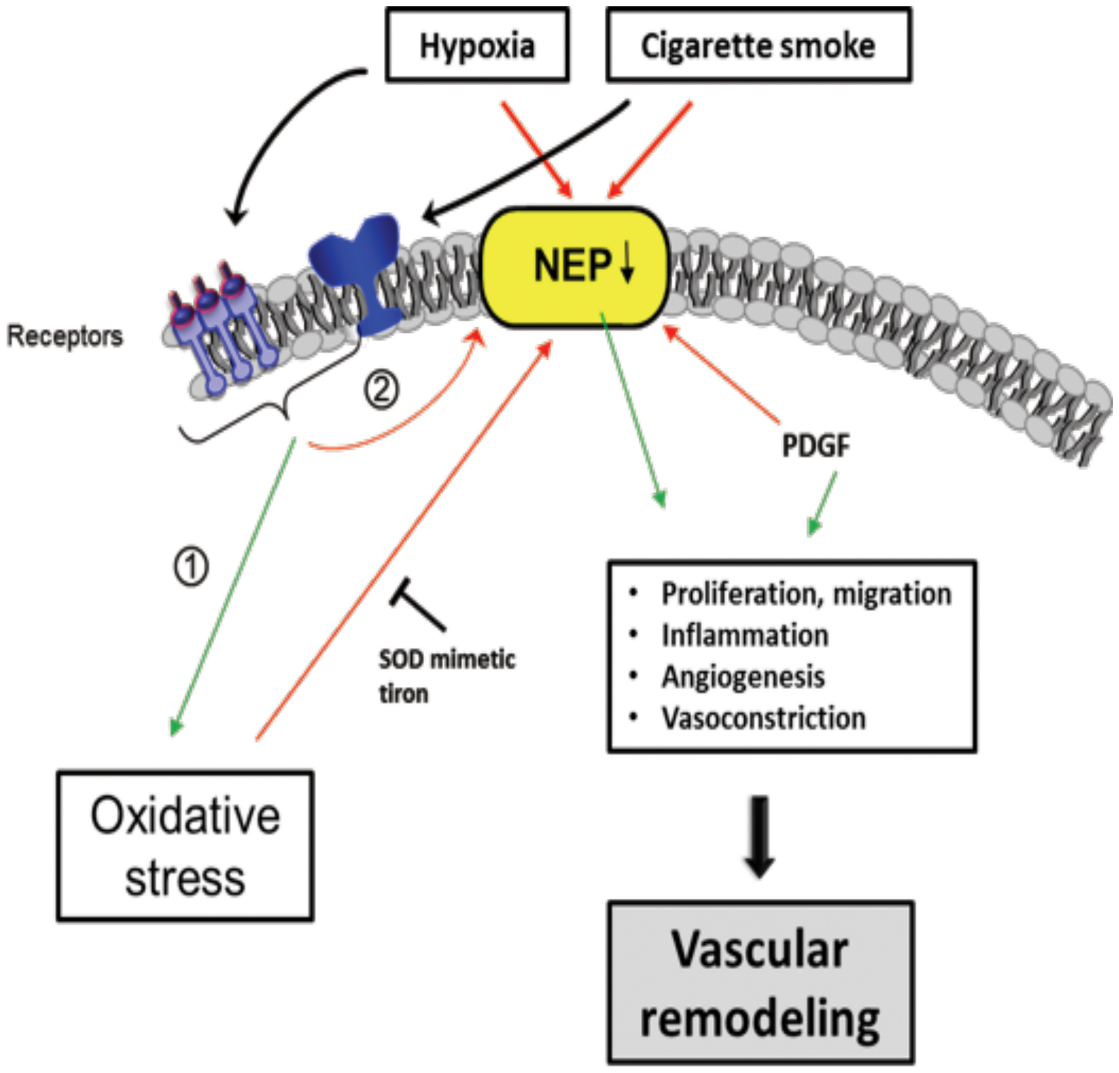
Scheme of proposed Neprilysin (NEP)-dependent mechanisms leading to vascular remodeling. Extracellular stimuli, such as hypoxia and cigarette smoke activate pathways in vascular cells causing downregulation of NEP expression and/or activity which was seen in patients with COPD associated with PH. Stimuli can have direct negative effects on NEP, but also indirectly by (1) increasing ROS, PDGF and (2) activation of other pathways, in part receptor-mediated. NEP downregulation leads to elevated proliferation, migration, inflammation, Angiogenesis, and vasoconstriction mediated amongst others by depicted molecules causing vascular remodeling. The ROS effect on NEP can be inhibited by the superoxide dismutase (SOD) mimetic tiron. Colored boxes indicate involvement in the respective pathway; red arrows, inhibition/downregulation; green arrows, activation/upregulation; PDGF, platelet-derived growth factor; ET-1, endothelin-1; FGF-2, fibroblast growth factor; AT-II, angiotensin-II; PASMC, pulmonary artery smooth muscle cells; EC, endothelial cells; FB, fibroblasts.

Neprilys in (neutral endopeptidase, NEP) may be an important factor for the regulation of susceptibility to pulmonary vascular remodeling in response to smoke inhalation and hypoxia (235) (Figure 6). NEP is a transmembrane zinc peptidase that is widely expressed in PASMCs, ECs, and fibroblasts (236). NEP expression and activity is decreased by CS (237), hypoxia (238, 239), and oxidative stress (240). The depletion of NEP in mice resulted in increased severity of PH, associated with greater proliferation of PASMCs. Therefore, it has been suggested NEP plays a protective role against PH, partly by suppressing the proliferation and migration of PASMCs (239).

Wick et al. found that NEP expression decreased in the lungs of COPD with PH as well as in non-COPD PH patients (235). NEP is involved in many peptidase-dependent (e.g., degradation of vasoactive neuropeptides) and -independent (e.g., interaction of signaling molecules with the intracellular cytosolic domain of NEP) signaling pathways (241, 242), but its role affecting vascular remodeling remains to be elucidated. In this regard, Wick et al. suggested that the proliferation/migration of dedifferentiated SMCs or myofibroblasts promotes pulmonary vascular remodeling and PH if NEP is less active or downregulated. It is assumed that this process is mediated by platelet-derived growth factor (PDGF), the expression of which is inversely correlated with NEP. Karoor et al. supported this assumption by showing that PDGF receptor (PDGFR) signaling was constitutively active in NEP^−/−^ cells and in lungs, an effect that is attenuated by the endothelin A (ET_A_) receptor antagonist ambrisentan (243). The decrease in NEP following CS and hypoxia may also enhance the angiogenic effect of fibroblast growth factor-2 (FGF-2) (242) and the pro-proliferative and vasoconstrictive responses of ET-1 (244) and bombesin-like peptides (245), which are substrates of NEP.

In PASMCs, both FGF-2 and ET-1 were shown to synergize with PDGF in increasing the phosphorylation of Src kinase and PDGFR (243). The phosphatase PTEN (phosphatase and tensin homolog) was also shown to play an important role in vascular biology, because a loss of PTEN results in PH (246). PTEN is inactivated by phosphorylation (mediated by Src and PDGFR) and downregulated in NEP-deficient PASMCs. This downregulation can be rescued by NEP overexpression in NEP null cells or by a reduction in Src or PDGFR by small interfering RNA (siRNA). Accordingly, it has been suggested that NEP-dependent mechanisms may protect against the inactivation of PTEN (243). In addition, NEP can be inactivated by ROS, as shown by its decreased activity in the presence of H_2_O_2_ and improved activity when an antioxidant, the SOD mimetic Tiron, was added (235).

Early studies suggested that the inhibition of NEP exerted beneficial effects in the treatment of PH (247, 248). This idea resulted from the fact that NEP can inactivate atrial/brain natriuretic peptides (ANP/BNP) that promote vasodilation by increasing cGMP, mediated by natriuretic peptide receptor-A (NPR-A) (248). cGMP-dependent protein kinase (PKG), cGMP binding phosphodiesterases (PDEs), and cyclic nucleotide-gated ion channels bind cGMP, with PKG seeming to be the main mediator of cGMP signals (249, 250). Binding of ANP/BNP-induced cGMP activates PKG, followed by the catalytic transfer of phosphate from ATP to target proteins. The phosphorylated proteins then translate the extracellular stimuli into specific biological outputs (251), such as vasodilation.

NEP antagonists alone and in combination with ACE (angiotensin converting enzyme) and ECE (endothelin converting enzyme) inhibitors were able to improve cardiac function, limit cardiac hypertrophy, and decrease systemic blood pressure (252–255). Nevertheless, side effects were observed if single NEP inhibitors or dual inhibitors (NEP/ACE or NEP/ECE) were used. Triple vasopeptidase inhibitors (NEP/ACE/ECE) showed promising preliminary results, with fewer side effects. In particular, an increase in ET-1 can be antagonized by simultaneous application of an ECE inhibitor (255). Combination therapies such as these, and the different mechanisms of NEP, ACE, and ECE inhibition, were reviewed by Daull et al. (255).

The existing literature supports the notion that NEP protects against PH (235, 239, 256). The discrepancy between beneficial and harmful pulmonary effects with NEP inhibition might be because pulmonary and systemic circulation usually respond to hypoxia (a major stimulus for PH) by divergent pathways: while pulmonary vessels contract to redirect blood flow to better oxygenated areas of the lung, systemic vessels dilate to increase the flow of oxygenated blood to areas of tissue hypoxia or ischemia (239). In conclusion, in terms of the lung, it is suggested that NEP be increased to treat PH, whereas cardiac NEP inhibition could be used for the treatment of hypertrophy and improvement of cardiac function. These studies provide insights into distinct regulation of NEP in cardiovascular disease and PH. Additional investigation are required whether therapeutic activation of NEP selectively in lung ameliorates PH.

## Stem Cells and Vascular Regeneration in Copd/Emphysema

The regeneration or replacement of structurally impaired lung tissue would represent a breakthrough in the treatment of pulmonary diseases, including COPD. Therefore, stem and progenitor cells may be a promising therapeutic approach for COPD. Various types of stem cells, including mesenchymal stem cells (MSCs), induced pluripotent stem cells (iPSCs), embryonic stem cells (ESCs), and stem cells derived from lung tissue, were tested in animal models and in a limited number of clinical studies as COPD treatment options (257–259).

**Figure 7:**
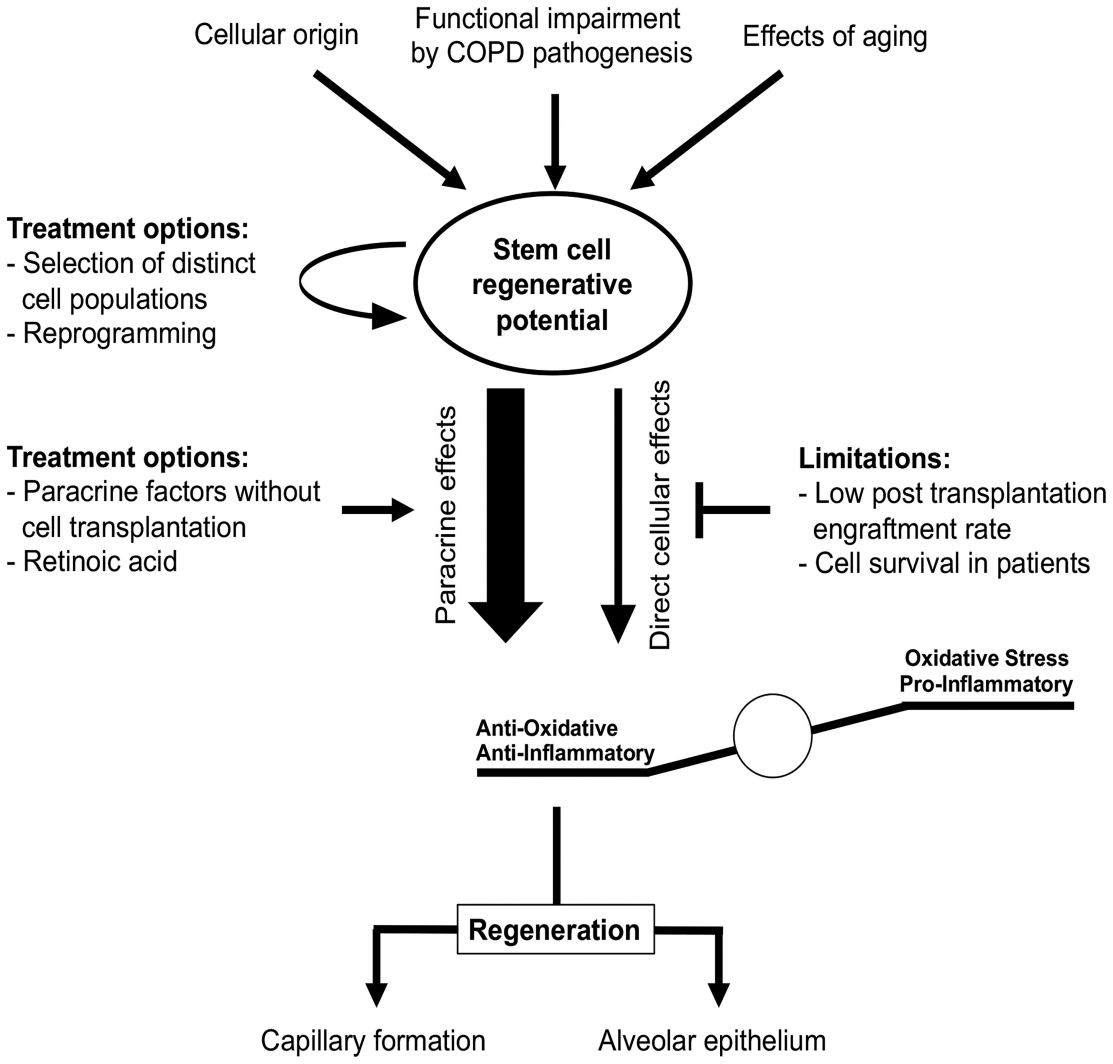
Stem cells in COPD treatment. Stem cell regenerative potential critically depends on cellular origin and can be impaired by different processes, e.g., due to aging or COPD itself. These impairments might be overcome by selection of distinct stem cell sub-populations or cellular reprogramming prior to transplantation. Regenerative potential of stem cells in COPD is rather mediated by paracrine effects reducing oxidative stress and pro-inflammatory stimuli than direct cellular effects, since cell engraftment and survival rates observed *in vivo* are low. Studies of COPD treatment have to focus not only on the functional regeneration of alveolar epithelium, but also on vascular aspects as both are required for effective therapy.

For the efficient treatment of COPD, regeneration of alveolar epithelium as well as capillary formation is required. Multiple stem cells have been shown to differentiate into ECs capable of capillary formation, both *in vitro* and *in vivo* (257, 260–263). Transplantation of pre-differentiated ESCs and iPSCs reduced lung injury in a bleomycin mouse model, presumably by reducing inflammation (257, 264). MSC transplantation was shown to protect against EC apoptosis in animal emphysema models and promoted restoration of both alveolar and endothelial structures (265–267). Furthermore, MSC-conditioned medium restored endothelial barrier impairment caused by CS (268). Li et al. reported that MSCs derived from iPCSs were superior to bone marrow-derived MSCs regarding CS-induced pulmonary airspace enlargement (258). This indicates that stem cells of different origin vary in their pulmonary regenerative potential. In contrast with these beneficial effects, the engraftment of transplanted cells has been found to be quite low (<2%) in murine models of lung emphysema (269–271). This is in accordance with a study by Huh et al., who reported that not only bone marrow cells or MSCs (which largely disappeared in pulmonary tissue over time post-transplantation) but also cell-free conditioned media obtained from MSCs alleviated CS-induced emphysema (272). This indicates a paracrine effect rather than a direct effect of stem cell engraftment and could explain the positive effects observed in studies with low stem cell engraftment rates (Figure 7). Therefore, it remains questionable how long stem cells engrafted after transplantation remain viable in patients. Moreover, this raises the question whether efficient effects in patients might require recurring cell transplantations.

At present, identifying the beneficial underlying paracrine mechanisms of stem cell effects in pre-clinical COPD models might reveal new treatment options without the need for cellular transplantation (Figure 7). Based on current studies, the beneficial paracrine effects of stem cells in COPD appear to be mediated by reduced inflammation and oxidative stress (269, 273, 274). Using these paracrine effects for cell-free therapy is clinically relevant, since ESCs and iPSCs can induce teratoma formation post-transplantation (275–277). This currently limits the utility of these cells in clinical studies. Furthermore, recent studies indicate that dissemination of cells or pre-differentiation of iPSCs in lung progenitor cells might overcome this problem (278, 279). However, this must be confirmed prior to the first clinical implementation.

Low engraftment rates and survival of transplanted stem cells might be one of the reasons for the failure of the first studies of clinical stem cell transplantation in COPD. Cell transplantation had no significant impact on pulmonary functions or exacerbations. However, circulating c-reactive protein was reduced in some studies, although for a limited time (280, 281). In other clinical studies, the number of patients included were either too low for sufficient interpretation (282) or the improvements in FEV1 were attributable to lung volume reduction surgery (283). Nevertheless, these studies provide substantial evidence for the safety of MSC transplantation in humans and thereby the basis for future studies.

It has been suggested that patients with advanced stages of COPD who are included in clinical studies might display pulmonary damage too severe to be reversed or repaired by transplanted cells (280). Therefore, patient inclusion criteria will be important for successful study protocols in the future. In addition, aging bone marrow-derived stem cells from animals and humans show impaired proliferation, decreased differentiation potential, and secretion of paracrine factors (284, 285). However, these age-dependent changes seem not to affect the entire stem cell population, as some subpopulations retain a more “youthful” phenotype (286). Therefore, pre-selection and perhaps reprogramming of suitable cells also needs to be considered in future studies. In this context, reprogramming of epigenetic modifications in COPD lung fibroblasts *in vitro* through an iPSC intermediate state was shown to result in fibroblasts similar to fibroblasts from non-COPD patients, indicating a possible treatment option without cellular transfer (287). Furthermore, the vitamin retinoic acid is important for pulmonary development, e.g., for progenitor cell differentiation, and was shown to be beneficial in animal emphysema models (288–293). Although clinical evidence is lacking (294), the reprogramming or activation of intrinsic stem and progenitor cells suggests an additional therapeutic treatment option.

Recent studies provide evidence that EPCs play a critical role in COPD. Circulating endothelial progenitor cell reduction in COPD is correlated with disease severity and inversely correlated with the extent of emphysema, whereas hematopoietic progenitor cells are unaltered (295–297). Intratracheal application of bone marrow-derived EPCs attenuated the development of pulmonary emphysema in mice exposed to CS in a long term murine study, *via* the alleviation of inflammatory infiltration, decreased proteolytic enzyme activity, and improved antioxidant activity (298). In contrast, enhanced progenitor cell numbers in pulmonary arteries obtained from patients with COPD were associated with decreased endothelium-dependent dilation and inversely correlated with arterial lumen area (299). However, the characterization of these cells by progenitor marker expression was limited in this study. As the level of circulating EPCs is decreased but their migratory potential and adhesion is increased in COPD, it was suggested that circulating ECs are recruited to pulmonary tissue in COPD (296, 299, 300). This might not be beneficial in COPD patients, since circulating progenitor cells in these patients show impaired angiogenic ability, increased apoptosis, and impaired NO production compared with these features of EPCs obtained from healthy, non-smoking controls (301, 302). The beneficial effects of endothelial progenitor cell transplantation obtained by Shi et al. (298) might at least partially be attributed to the non-COPD origin of stem cells in this study. Therefore, modulation of EPCs in COPD *in vivo* or pre-transplantation to re-achieve physiological cell functions might be a promising approach in the treatment of COPD.

## Conclusion

Taking the above evidence together, the mechanisms underlying COPD and PH are still not fully understood. However, experimental studies and clinical observations have mechanistically linked vascular dysfunction with the development of COPD. Vascular remodeling and PH can occur in cases of COPD, not only in severe cases but also in mild-to-moderate forms of the disease and even in smokers with no airflow limitations. COPD associated with PH and pulmonary vascular remodeling is a multifactorial disease which involves hypoxia-related, hypoxia-unrelated, inflammatory, and endothelial dysfunction-associated mechanisms. Recent investigations have changed the view of the pathophysiology of COPD. In the past, vascular remodeling was suggested to be a secondary event occurring following the destruction of the parenchyma, with the predominant causes being hypoxia and hypoxemia. This perception has changed, with recent observations demonstrating that vascular abnormalities can be early events in COPD, which precede airflow limitations and emphysema independent of hypoxia. PH and lung emphysema can occur independently, suggesting that vascular molecular alterations can be a trigger for the development of lung emphysema. In this regard, the NO–sGC–cGMP axis might play an important role in both pathology and regenerative potential. However, further investigations of the contribution of pulmonary vascular changes to the development of COPD are needed to identify new therapeutic targets for this disease. The endothelium is known to regulate homeostasis between vasoconstriction and vasodilation, providing adequate perfusion pressure. However, exposure to toxic radicals can lead to ED *via* leukocyte infiltration and adhesion, resulting in tissue injury. Thus, ED changes the permeability of tissues and leads to structural damage of arterial walls *via* the proliferation of SMCs, resulting in CVD (303, 304). Cigarette smoke-induced CVD reduces the regenerative capability of the cardiovascular system; however, EPCs modulate vasculogenesis. EPCs modify cardiovascularity *via* the release of cytokines, vascular growth factors, and chemokines, thereby promoting transdifferentiation of cardiac SMCs and reducing the risk of CVD (305–307). Stem cell-based therapeutic strategies should therefore be investigated for the treatment of ED in PH and CVD-associated COPD patients.

## Author Contributions

SK, MS, FK, AS, TM, AB, DK, and KK conceived the focus of the review, wrote, and drafted the paper. NW and SE contributed intellectual content and expertise. SK, MS, and FK prepared the figures. SK supervised the study. All authors listed have made a substantial, direct and intellectual contribution to the work, and approved it for publication.

## Conflict of Interest

The authors declare that the research was conducted in the absence of any commercial or financial relationships that could be construed as a potential conflict of interest.
